# From Bench to Bedside: Motor–Cognitive Interactions

**DOI:** 10.3390/brainsci14090886

**Published:** 2024-08-30

**Authors:** Daniele Corbo

**Affiliations:** Department of Medical and Surgical Specialties, Radiological Sciences and Public Health, University of Brescia, 25123 Brescia, Italy; daniele.corbo@unibs.it

## 1. Introduction

The capacity of humans to learn new motor abilities is known as motor learning, which is often understood as increasing movement precision over time and space through repetition [1,2,3]. Theories suggest that motor learning involves cognitive processes such as working memory and is not only a physical function [4,5,6]. Although motor and cognitive deficits are often studied separately, a connection between the two is emphasized in our growing understanding of the task-dependent interaction between the motor and cognitive systems, each of which has different neuroanatomic substrates [7,8,9,10]. The importance of motor–cognitive interactions in neurodegenerative illnesses and other clinical groups, including dementia, stroke, Parkinson’s disease, and multiple sclerosis, is becoming increasingly evident. As such, considerable effort has been devoted to creating rehabilitative procedures that focus on motor–cognitive connections to address the circumstances associated with these disorders. 

The ten papers and six reviews in this Special Issue of *Brain Sciences* provide an intriguing and well-matched mixture of all these research areas in terms of understanding the fundamental processes of motor–cognitive connections and novel therapies. The first group of papers focuses on experimental and clinical investigations dealing with relevant aspects of the motor–cognitive interactions, and a second group of articles consists of review papers, which collate the existing literature on various aspects concerning the connections between motor skills and neural effects and their possible clinical applications. The articles in this collection, based on the topics covered, can be classified into three sugroups: mechanisms of cognitive–motor interactions, diagnostic tools, and intervention strategies.

## 2. Cognitive–Motor Interaction Mechanisms

Xiao et al. (contribution 1) used a mouse-tracking technique to analyze the hand motions of participants to investigate the role of attention in subliminal semantic processing. Their findings suggest that the temporal–spatial features extracted from cursor motion trajectories can reliably reveal subliminal semantic processing and attentional status, proving that, for a wide range of topics, the mouse-tracking approach is a suitable tool for uncovering implicit dynamic cognition in future studies.

In an investigation into the brain mechanisms underpinning the perception of others’ activities, Urgen et al. (contribution 2) provided a series of videos featuring 100 human behaviors captured in real environments. The study observation of the 100 events triggered a well-established action observation network, and they used fMRI to validate the dataset. This extensive collection of videos is a valuable tool for studying the brain and perception.

Dahm et al. (contribution 3) investigated whether leg vs. arm left–right judgments are harder and if limb type affects these judgments. A combined score for accuracy and speed was investigated to further avoid any trade-offs and accurately assess each subject’s unique ability. They concluded that realistic stimulus material enhances the effects of perspective and facilitates the understanding of tasks. The linear speed–accuracy score was found to be a reliable indicator of performance in mental body rotations by repeating earlier research findings.

The flexibility of the peripersonal area was examined by Ferroni et al. (contribution 4) both before and after actual or virtual motor training using a tool. Their findings demonstrate that the peripersonal area only expands in response to the use of real-world tools, not in response to virtual ones, underscoring the possibility that the two forms of training depend on distinct mechanisms. The state of the art regarding the malleability of the peripersonal area in both real and virtual environments is enhanced by this study. The authors discuss about their data’s applicability to the creation of training, learning, and rehabilitation immersive environments.

Van Hove et al. (contribution 6) aimed to examine how speech production and cognitive load levels affect timed up and go (TUG) and static equilibrium tasks. They assessed the impact of speech production (SP), cognitive load (CL), and dual-task cost (DTC) on these variables. The center of pressure oscillation velocity during static balancing was substantially higher when both tasks were completed orally than in the control scenario. The cognitive load was linked to an increase in TUG, but the oral or mental component did not appear to have any impact. SP more strongly impacted mobility in complicated cognitive tasks. This might be crucial for test selection and comprehending abnormalities in postural control.

## 3. Diagnostic Tools

The discriminating value of the trail-walking test was assessed by Klotzbier et al. (contribution 5) as a prospective diagnostic tool to enhance the prediction ability during clinical evaluation regarding the severity of Parkinson’s disease and documenting the many walking-related characteristics. Patients with Parkinson’s disease who exhibit postural instability or problems with walking must be diagnosed using reliable, thoroughly assessed clinical tests that provide an accurate evaluation of each patient’s unique fall risk, illness severity, and disease prognosis. The trail-walking test allows for the distinction of motor phenotypes in Parkinson’s disease and covers a variety of mobility-related topics, including the link between walking and cognitive functioning.

Chen et al. (contribution 7) created a prediction model of cognitive degradation in patients with Parkinson’s disease using a machine learning technique. The clinical information, plasma biomarkers, and cognitive test results of people with Parkinson’s disease were gathered as model predictors. Machine learning techniques such as principal component analysis and support vector machines were used to create a cognitive categorization model. The classifier achieved an accuracy of 92.3% and an area under the receiver operating characteristic curve (AUC) of 0.929 employing 32 comprehensive predictive criteria. Furthermore, with 13 well-selected features, the accuracy of the classifier was increased to 100% and the AUC to 1.0. The priorities for future perspectives include expanding the sample size and conducted a longitudinal investigation.

Beauchet et al. (contribution 8) investigated the relationship between incident major neurocognitive disorders (MNCDs) in older community-dwelling individuals and the inability to name the date (a sign of cognitive impairment), the use of a walking assistance, and/or a history of falls (a sign of motor impairment). The incidences of MNCDs was found to be higher when the inability to recall the date and the use of a walking assistance and/or a history of falls were combined. This suggests that the combination of items may be used for screening for the risk of MNCD in the older population, particularly for the incidence of AD. This study opens new avenues for identifying MNCD risk and managing its modifiable risk factors due to the ease with which older populations may obtain data on both of these factors.

Corbo et al. (contribution 9) trained and evaluated a unique automated and image-derived scoring system to improve the capability to discriminate the sensorimotor impairments that are predictive of sensorimotor dysfunction with the Luria–Nebraska Neuropsychological Battery (LNNB) for neuro-motor tasks. The conventional scores, which were evaluated and verified by numerous administrators to reduce subjectivity, showed a strong association with the image-derived LNNB task scores (Pearson’s correlation > 0.70). The innovative image-based scoring method distinguished between individuals with poor motility (<mean population values) with 70–83% specificity and 70% sensitivity. The new image-derived LNNB task scores have potential for use in telemedicine and in the timely evaluation of sensorimotor skills and delays.

Chen et al. (contribution 10) investigated the variables linked to the fear of falling (FOF) in people with moderate cognitive impairment (MCI) owing to Parkinson’s disease (PD-MCI) and minor cognitive impairment (MCI) owing to Alzheimer’s disease (AD-MCI). In the AD-MCI group, the FOF was strongly linked with gait speed, stride length, Tinetti assessment scale score, executive function, attention and working memory, and global cognitive function. Furthermore, the primary causes of the FOF were working memory and attention. The FOF strongly correlated with both gait speed and timed up and go subtask performance in the PD-MCI group. Moreover, the primary cause of the FOF was turn-to-walk behavior. Therapies targeting attention and working memory and turn-to-walk, respectively, may be used to reduce the FOF in people with AD-MCI and PD-MCI.

## 4. Intervention Strategies

The review of Saviola et al. (contribution 11) summarizes the research on the neuroimaging outcomes of physical therapy in cohorts of patients with psychosis. The twenty-one studies included in this narrative review were all research publications. Saviola et al. suggest that physical intervention is now considered the standard for helping patients with psychosis experience brain alterations. This means that physical intervention is beneficial not only when the disease first manifests but also for enhancing the illness’s course and functional result. However, additional data are required to further the understanding of the long-term plastic reorganization of the psychotic brain, particularly in areas of the brain that have not been thoroughly studied, including motor circuits.

Xiao et al. (contribution 12) summarized the available data on the impact of dual-task training on motor and cognitive skills in patients with Parkinson’s disease to support the therapeutic practice of dual-task training. The present views on the mechanism underlying the interplay between motor and cognitive training were also covered. In summary, dual-task training can help people with PD with varying lengths of illness to enhance their motor performance. Dual-task training can help with balance, single-task steep length, single-task gait speed, objective experience of gait freezing in Parkinson’s disease, and motor symptoms. This review has several restrictions as well: Because the control intervention and dual-task training design differed among the studies, studies that were not written in English were excluded. Additionally, study quality varied because both RCT and non-RCT studies were included.

Deste et al. (contribution 13) conducted an overview of the literature on physical exercise as a treatment for cognitive impairment in schizophrenia and of the studies that combined physical exercise and cognitive remediation as an integrated rehabilitation intervention. More research is currently required to better understand how to incorporate physical exercise and cognitive remediation in psychiatric rehabilitation practice, even though these interventions seem to be effective treatments for cognitive impairment in people with schizophrenia.

Pertichetti et al. (contribution 14) systematically reviewed the scientific literature on both neuropsychological tests and fMRI tasks for preoperative planning. Changes in functions during the neuropsychological evaluation may assist in identifying patients who can benefit from fMRI and, potentially, functions that should be examined, according to the correlation between the findings of the two tests. fMRI and neuropsychological testing play complimentary roles in the preoperative evaluation. The small number of studies that satisfied the inclusion criteria is the main constraint of this study. This dearth of information is a reflection of the diversity of the literature in terms of behavioral experimental design, neuropsychological testing, fMRI investigations, and particular objectives examined.

Kamińska et al. (contribution 15) assessed the efficacy of different treadmill training outcomes in individuals with Down syndrome (DS), including adults and children. With a total of 687 people, they chose 25 trials for analysis and found 25 distinct results, which are then narratively presented. They found favorable benefits in every case, with the treadmill training being the most effective. People with DS see improvements in their physical and emotional health when they incorporate treadmill exercise into their regular physiotherapy regimen.

Jylänki et al. (contribution 16) conducted a systematic review to better understand the methodological quality and the impact of physical exercise and fundamental motor skill therapies on academic and cognitive skills in 3- to 7-year-old children with special educational needs. The effects of the intervention seemed to vary depending on the severity of the learning difficulty. Regarding language and cognitive skills, children who were at risk because of their family background benefited the most from the intervention, whereas children with learning disabilities benefited most in terms of executive functions. However, providing a broadly applicable summary of the results is difficult because of the wide variation in the included studies and the relatively low methodological quality. Therefore, more thorough studies are needed to evaluate the efficacy of these therapies.

Taken together, the papers gathered in this Special Issue of *Brain Sciences* dealing with motor–cognitive interactions should therefore be of considerable interest for neuroscientists interested in understanding of key mechanisms of motor–cognitive interactions and innovative treatments.
